# The Safety and Efficacy of the Combination of Sacituzumab Govitecan and Palliative Radiotherapy—A Retrospective Multi-Center Cohort Study

**DOI:** 10.3390/cancers16091649

**Published:** 2024-04-25

**Authors:** David Krug, Joke Tio, Ali Abaci, Björn Beurer, Sandra Brügge, Khaled Elsayad, Eva Meixner, Tjoung-Won Park-Simon, Katharina Smetanay, Franziska Winkelmann, Andrea Wittig, Achim Wöckel

**Affiliations:** 1 Department of Radiation Oncology, University Hospital Schleswig-Holstein, 24105 Kiel, Germany; 2 Department of Gynecology and Obstetrics, Section Senology, University Hospital of Muenster, 48149 Muenster, Germany; joke.tio@ukmuenster.de; 3 Department of Radiotherapy, Hannover Medical School, 30625 Hannover, Germany; abaci.ali@mh-hannover.de; 4 Department of Obstetrics and Gynecology, Ernst von Bergmann Clinic, 14467 Potsdam, Germany; bjoern.beurer@klinikumevb.de; 5 Department of Gynaecology and Obstetrics, University Hospital Schleswig-Holstein, 24105 Kiel, Germany; sandra.bruegge@uksh.de; 6 Department of Radiation Oncology, University Hospital of Muenster, 48149 Muenster, Germany; khaled.elsayad@ukmuenster.de; 7 Department of Radiation Oncology, University Hospital Heidelberg, 69120 Heidelberg, Germany; eva.meixner@med.uni-heidelberg.de; 8 Department of Obstetrics and Gynecology, Medizinische Hochschule Hannover, 30625 Hannover, Germany; park-simon.tjoung-won@mh-hannover.de; 9 National Center for Tumor Diseases and Department of Obstetrics and Gynecology, University Hospital Heidelberg, 69120 Heidelberg, Germany; katharina.smetanay@med.uni-heidelberg.de; 10 Department of Radiation Oncology, Ernst von Bergmann Clinic, 14467 Potsdam, Germany; franziska.winkelmann@klinikumevb.de; 11 Department of Radiotherapy and Radiation Oncology, University Hospital of Wuerzburg, 97080 Wuerzburg, Germany; wittig_a1@ukw.de; 12 Department of Obstetrics and Gynecology, University Hospital of Wuerzburg, 97080 Wuerzburg, Germany; woeckel_a@ukw.de

**Keywords:** breast cancer, antibody–drug conjugate, brain metastases, radiosurgery, stereotactic radiotherapy, palliative radiotherapy

## Abstract

**Simple Summary:**

Sacituzumab govitecan is an antibody–drug conjugate that has been approved for the treatment of metastatic triple-negative breast cancer and, recently, also for metastatic hormone receptor-positive HER2-negative breast cancer. Although patients with metastatic breast cancer frequently need palliative radiotherapy, none of the published prospective trials studied the combination of radiotherapy and Sacituzumab govitecan. We conducted a multi-center retrospective cohort study to assess the efficacy and safety of Sacituzumab govitecan and palliative radiotherapy. We did not find signs of increased toxicity, and response rates were favorable despite extensive prior treatment. More research is necessary to determine the optimal integration of novel systemic agents and radiotherapy in the palliative setting.

**Abstract:**

Sacituzumab govitecan (SG) is a new treatment option for patients with metastatic triple-negative and hormone receptor-positive, HER2-negative breast cancer. This antibody–drug conjugate is currently approved as monotherapy. Palliative radiotherapy is frequently used to treat symptomatic metastases locally. Concurrent use of SG and irradiation was excluded in clinical trials of SG, and there are currently limited published data. We report here a systematic review, as well as a retrospective multi-center study of 17 patients with triple-negative breast cancer who received concurrent SG and radiotherapy. In these patients, concurrent use was found to be efficient, safe and well tolerated. There were no apparent differences in moderate or severe acute toxicity according to the timing of SG administration.

## 1. Introduction

Triple-negative breast cancer (TNBC) is a heterogeneous, aggressive tumor that often recurs after 2–5 years and is associated with a poor prognosis [1]. Due to the lack of expression of hormone receptors and HER2, therapeutic options are limited. Many tumors neither display PD-L1 (Programmed Cell Death Ligand 1) expression nor are BRCA (Breast Cancer)-1/2 mutations present, rendering these patients ineligible for checkpoint or PARP (Poly (ADP-ribose)-Polymerase) inhibitors in the metastatic setting [2]. Therefore, treatment options are currently limited to palliative chemotherapy in most patients with metastatic TNBC, and Sacituzumab govitecan (SG) is a much-needed option as an alternative to taxane- or platinum-based chemotherapy.

SG is a first-in-class antibody–drug conjugate (ADC). The monoclonal antibody targets the human trophoblast cell-surface antigen (Trop-2), which is overexpressed on approximately 90% of triple-negative breast cancers (TNBCs) [3,4]. Upon binding, the ADC delivers its payload SN-38, the active metabolite of irinotecan. A phase III ASCENT trial with SG demonstrated a significant improvement over standard chemotherapy with respect to median progression-free survival (PFS) and median overall survival (OS) in patients with TNBC who had received at least two chemotherapy regimens for advanced disease [5]. The most frequent side effects included neutropenia, diarrhea, nausea, alopecia, fatigue and anemia. Based on these results, SG as monotherapy was approved by the FDA and the EMA for the treatment of adult patients with unresectable or metastatic TNBC who had received two or more prior systemic therapies, including at least one of them for advanced disease [6]. Recently, the approval was extended to patients with unresectable or metastatic hormone receptor-positive, HER2-negative breast cancer who had received endocrine-based therapy and at least two additional systemic therapies for advanced disease.

Since SG has been studied and approved as monotherapy, experience with the combined use with radiotherapy is very limited. In four prospective clinical trials of SG (ASCENT, IMMU-132-01, TROPHY-U-01, TROPiCS02), radiation therapy had to be completed ≥ 2–4 weeks prior to randomization, as described in the respective trial protocols [5,7,8,9,10].

Although there is no study currently addressing the role of the combined use of radiotherapy and SG, it is expected that many patients with metastatic TNBC will require palliative radiation at some point while receiving this drug due to local progression or local symptoms.

According to a recent meta-analysis, approximately one-third of patients with metastatic TNBC will eventually develop brain metastases [11]. In the ASCENT trial with SG, patients with stable brain metastases were eligible to enter the trial, but they were a small cohort (n = 61) and excluded from the primary analysis. Patients with active brain metastases were not eligible [5]. The main therapeutic approach for brain metastases in TNBC is local therapy, i.e., surgery, stereotactic radiosurgery (SRS), stereotactic radiotherapy (SRT), or whole-brain irradiation (WBRT), with the choice of therapy depending on the localization, size and number of metastases, as well as previous therapy, performance status and prognosis [12,13]. In many of these cases, the challenge of optimally combining systemic therapy with radiotherapy arises at some stage of the disease. Further indications for palliative radiotherapy in patients with breast cancer include symptomatic bone, skin or lymph node metastases.

A recent international multidisciplinary consensus on the combination of radiotherapy with targeted agents for breast cancer explicitly states the lack of evidence regarding the administration of SG with radiotherapy [14].

In this article, we summarize the available data on the combined administration of SG with radiotherapy, as well as the patient cohort treated in our centers with this combination. In these cases, we were interested in the feasibility, safety and tolerability of combined radiation and SG therapy and whether differences existed between concurrent and sequential administration.

## 2. Materials and Methods

### Search Strategy

A comprehensive literature search of peer-reviewed journals was performed in the Ovid MEDLINE^®^Biosis, Journals@Ovid and Citations Daily databases from 1946 to February 2024, BIOSIS Previews from 1993 to February 2024 and Embase from 1974 to February 2024. The search strategy was restricted to the English language, and publications on the combined use of SG and radiotherapy published between 1946 and February 2024 were selected. Specific search terms are found in Table 1. The Population, Intervention, Control, Outcome, Study Design (PICO) criteria and Preferred Reporting Items for Systematic Reviews and Meta-Analyses (PRISMA) Checklist can be found in the Supplementary Materials (Tables S1 and S2). The data were extracted by two authors (DK and AWö).

All participating institutions queried their databases for patients receiving SG for metastatic TNBC. The search was further limited to patients that received palliative radiotherapy. Concomitant radiotherapy was defined as the administration of SG within 90 days before or 90 days after radiotherapy. This included either simultaneous administration, defined as administration of SG during the course of palliative radiotherapy, or sequential administration. Patients were eligible irrespective of the location and indication for irradiation, dose schedule and fractionation. Data regarding the dose of SG; tolerability before, during and after radiotherapy; target volume; dose and fractionation of radiotherapy; toxicity; and prior and subsequent treatment lines were collected from electronic health records.

## 3. Results

### 3.1. Published Clinical Data on Sacituzumab Govitecan Combined with Radiotherapy

**Figure 1:**
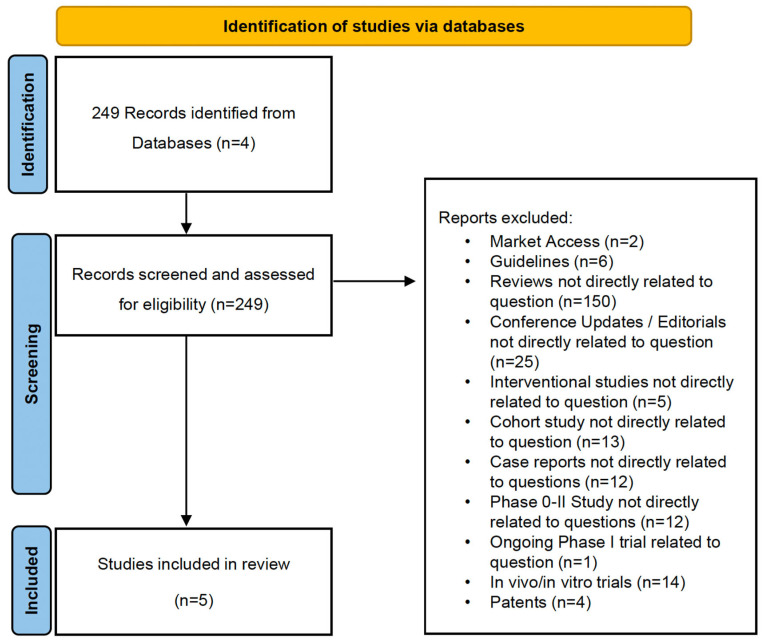
PRISMA chart adapted from [20].

The literature review of published data resulted in five publications, including one abstract on real-world experience with SG [15], three clinical case reports [16,17,18], and one single-center case series [19] [Figure 1].

Di Mauro et al. report on the case of a 59-year-old woman with a BRCA mutation and metastatic TNBC, who was treated with SG and radiotherapy [16]. The patient received SG (10 mg/kg on days 1 and 8 every 21 days) as palliative second-line treatment as part of an Expanded Access Program. Due to extensive central nervous system involvement, the patient received simultaneous whole-brain radiotherapy (WBTR), 30 Gy in 10 fractions, starting 2 days after day 8 of the first cycle of SG. Treatment with SG was restarted 8 days after the end of WBRT upon the patient’s request. The patient reported symptomatic relief after the first cycle of SG, and a subsequent CT scan after 3 months showed an extracranial partial response and a near-to-complete intracranial response. No grade 3 adverse events were reported, but SG was reduced to 7.5 mg/kg due to persistent grade 2 asthenia. After 10 months from starting SG, systemic disease progression was documented, while intracranial response was maintained [16].

McNamara et al. reported on a 68-year-old woman with metastatic uterine serous carcinoma [17]. In actuality, while the case report details the use of trastuzumab deruxtecan in this patient, it is briefly mentioned that the patient previously received SG and was treated with palliative radiotherapy while on SG. The authors state that SG was halted for several weeks before and after radiotherapy. Later scans demonstrated a partial remission of the disease after switching to trastuzumab deruxtecan. Local response to radiotherapy and potential toxicities are not mentioned.

Zhu et al. published their experience with three cases with brain metastases treated with CyberKnife or tomotherapy [18]. Among them, one patient with metastatic urothelial carcinoma received third-line therapy consisting of SG plus pembrolizumab. Subsequently, the patient was diagnosed with brain metastases and was treated with fractionated stereotactic radiotherapy to two lesions. Based on MRI one month after treatment and clinical assessment, the patient responded to the treatment. The timing of radiotherapy and SG administration and toxicity were not reported [18].

Hanna et al. reported on the treatment of 132 patients with SG; 11 of them received additional radiotherapy because of brain metastases [15]. Importantly, patients who received radiotherapy had superior PFS compared to patients that had no radiotherapy (hazard ratio 0.27, 95% confidence interval (CI) 0.1–0.71, *p* = 0.006). However, no treatment details regarding radiotherapy or information on toxicity were reported.

Lebow et al. recently published a single-center case series of 98 patients who received stereotactic radiotherapy for brain metastases and were also treated with ADCs, namely T-DM1, trastuzumab deruxtecan or SG [19]. There were 26 patients who received SG. Concurrent use was defined as the administration of radiotherapy either ≤7 days before or ≤21 days after ADC infusion. Patients who received concurrent ADC had a significantly higher risk of symptomatic radiation necrosis (sub-hazard ratio (sHR), 4.31, 95% CI 1.95–9.50; *p* < 0.001), especially in the setting of re-irradiation. There was no significant difference between the individual ADC in terms of radiation necrosis. In a response to comments, the authors reported that for SG, there was no significantly increased risk of symptomatic radiation necrosis (sHR 5.18, 95% CI 0.64–42.11, *p* = 0.12). Efficacy was not reported.

### 3.2. Results of the Multi-Center Retrospective Cohort Study

#### 3.2.1. Patient and Treatment Characteristics

A search of the institutional databases of the six participating centers revealed 17 cases of female patients who had been treated with SG and had received sequential or simultaneous palliative radiotherapy. The median age of the patients was 48 years (range 33–73 years), and the ECOG performance status was between 0 and 1 in most patients. They had received a median of 2.5 prior treatment lines [Table 2]. Additional patient and treatment characteristics can be found in the Supplementary Materials (Table S3).

**Figure 2:**
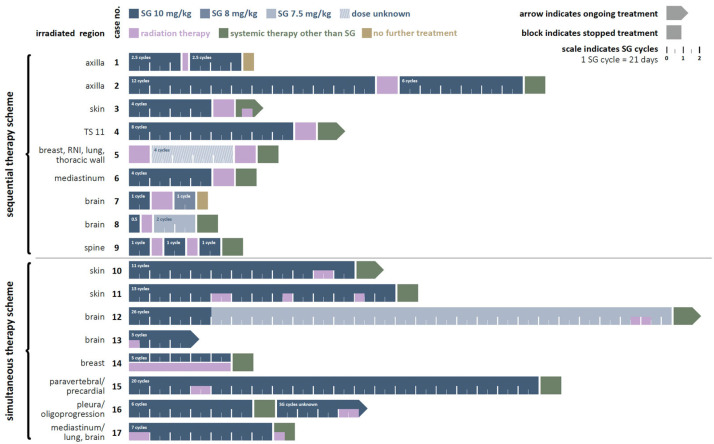
Overview of drug and radiation therapy of the described 17 cases. LN, lymph node; RNI, regional nodal irradiation; RT, radiotherapy; SG, Sacituzumab govitecan; TD, total dose; TS, thoracic spine.

All patients had triple-negative breast cancer (TNBC) at initial diagnosis or at the time of metastasis. Patients received a median of five cycles of SG, most of them at the approved dose of 10 mg/kg body weight. Further treatment details are shown in Figure 2.

Safety and tolerability data were available for 15 patients. Overall, SG was well tolerated. The most common adverse events were hematologic events, especially neutropenia, and fatigue. All patients treated with SG at our centers received G-CSF as a primary prophylaxis.

Other reported adverse events included infections (one case of herpes zoster, two cases of enterocolitis), diarrhea, abdominal pain, chest pain and alopecia. Fatigue grade 2 was reported in six cases after radiotherapy.

Response to SG treatment was assessed in 13 patients. Five patients initially responded to SG treatment, and two others had mixed responses, while six patients developed progressive disease on SG.

Of the seventeen patients, two patients were still on SG at the time of documentation, thirteen had received follow-up therapies (eribulin n = six, trastuzumab deruxtecan n = two, one each of capecitabine, niraparib and nab-paclitaxel/bevacizumab and unknown n = two). Two patients did not desire any further treatment after progression on SG. At the time of documentation, eleven patients were deceased.

#### 3.2.2. Radiotherapy

All but five patients had received radiotherapy during their prior course of disease before SG administration.

Seventeen patients received 34 local radiotherapy series in combination with SG. Eight patients received SG simultaneously to radiotherapy. SG and radiotherapy were administered sequentially in nine patients with a median time interval between SG and radiotherapy of 7 days (range 1–33 days). SG was resumed after a median of 6 days (range 1–68 days). In thirteen cases, treatment with SG was started first; in two cases, SG and radiotherapy were started simultaneously; and in one case, radiotherapy preceded treatment with SG [Figure 2]. The reason for concomitant radiotherapy in most patients was to treat a local progressive metastasis. Other reasons were the treatment of a local relapse (n = 1), palliation of symptoms or ulcerations (n = 2) or postoperative irradiation after resection of a brain metastasis (n = 1).

Radiotherapy details are shown in Table 2. In 12 radiation courses, there was an overlap with prior radiotherapy courses. The median time interval between combined SG/radiotherapy and prior radiation courses was two years (range, 1–7). The median cumulative radiotherapy dose was 79 Gy (range, 65–102).

#### 3.2.3. Toxicity and Treatment Outcome

**Figure 3:**
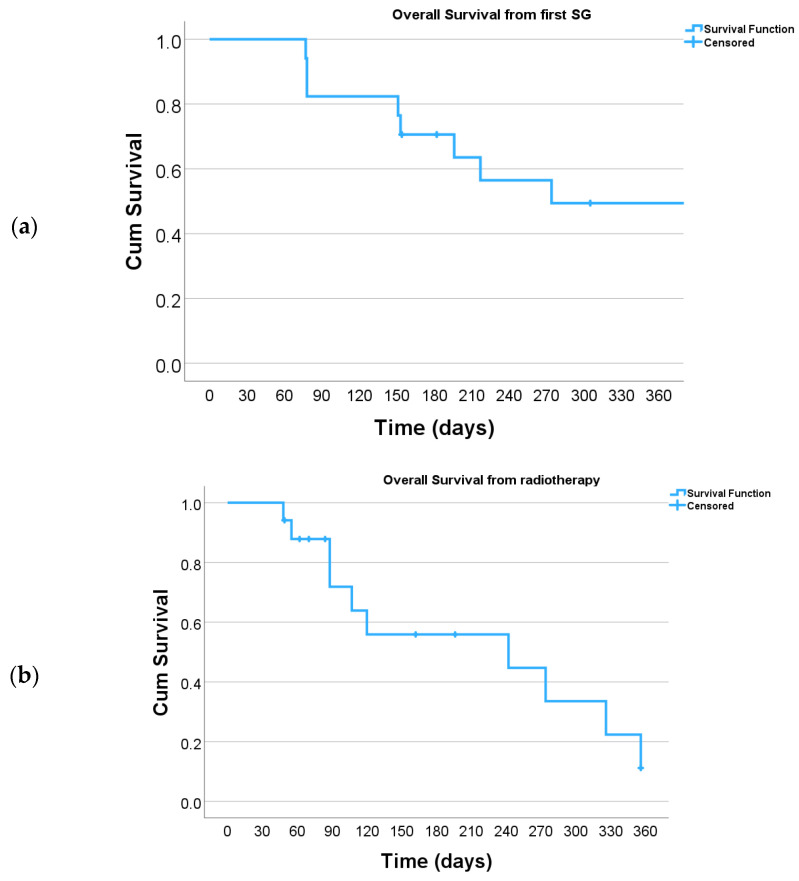
Kaplan–Meier plot of overall survival: (**a**) from first SG dose and (**b**) from end of radiotherapy.

Radiotherapy combined with SG was well tolerated without grade 4 or 5 acute or late toxicities. However, 82.3% of patients had toxicities. The most common toxicities were dermatitis and fatigue. In-field grade 3 toxicities were dermatitis (n = 2) and esophagitis (n = 1). Grade 3 toxicities occurred only in patients with overlap with prior radiation fields. Three patients (37.5%) with simultaneous administration and four patients (44.4%) with sequential administration suffered from acute grade 2–3 toxicity, respectively. At the first follow-up examination, 4 to 8 weeks after radiation therapy, the response rate per treatment course was 82.1%. In six lesions, the radiotherapy response was not available. Median overall survival after the first dose of SG was 217 days, ranging from 77 to 1080 days (i.e., 2.5 to 36 months). Median overall survival after the end of radiotherapy was 107 days, ranging from 48 to 356 days (i.e., 1.6 to 12 months) [Figure 3].

## 4. Discussion

Our systematic review demonstrates the lack of published clinical experience with the combination of SG and RT. Due to the small sample sizes, the retrospective design, the incomplete reporting of outcome and toxicity data and the lack of control groups, the risk of bias is high, and the quality of evidence is low.

In our own analysis, we demonstrate that the combination of SG and RT was not associated with unexpected treatment-related side effects and achieved a high response rate within the irradiated treatment volume despite extensive prior treatment and prior progression during SG in the majority of patients. Since many patients with metastatic breast cancer are in need of palliative radiotherapy for various reasons, especially local progression under systemic treatment, symptomatic metastases in bone and skin, brain metastases, bleeding or ulceration from the primary tumor, this is an important finding for clinical practice given the limited published data available.

When considering the combination of radiotherapy and systemic therapy, it is important to assess both efficacy and safety. While there are many established clinical situations where radiotherapy and systemic therapy are intentionally combined to enhance the effect of radiotherapy, i.e., chemoradiotherapy with drugs such as platinum salts, taxanes or 5-fluorouracil, only a minority of antineoplastic substances are primarily approved for combination with other therapeutic modalities [21]. In addition, systemic therapies are rarely developed specifically as radiosensitizers. Clinical decision making is challenging for the treatment of patients receiving systemic treatments with newly approved drugs that have never been systematically tested in combination with radiotherapy. In these cases, treating physicians cannot weigh the risks and benefits based on any evidence. Due to the high number of newly approved substances per year, this clinical problem is becoming increasingly important.

It is important to recognize that the risk profile of concomitant radiation with systemic therapies changes with the anatomic site being irradiated and the corresponding organs at risk in that location [22], treated volume, dose, fractionation and classes of substances of the systemic treatment.

When considering the potential toxicity of an ADC, the monoclonal antibody component as well as the cytotoxic payload have to be taken into account. One should therefore exercise caution when combining these drugs with radiation, given that a potentially radiosensitizing payload and other effects from the antibody conjugate may exacerbate radiation toxicity [23]. SN-38, the payload of SG, is a topoisomerase I inhibitor and a moderate radiosensitizer that increases the effect of irradiation by 1.5- to 2-fold [24]. Mechanistically, the inhibition of DNA repair by topoisomerase inhibitors has been well studied [25,26].

Antibody targeting can achieve significantly higher cytostatic drug concentrations in tumor tissue. This also potentially increases radiosensitization, and resistance development could be reduced. However, the side effects of combined treatment could also increase, particularly in normal tissues that express Trop-2. These are various epithelia of the skin, esophagus, lung, liver and other organs [27,28]. Alhough the half-life of SN-38 is only 14 h, the monoclonal antibody clears more slowly with a half-life of 5 days [29]. Since diarrhea is one of the most common toxicities of SG, radiotherapy with a significant dose to gastrointestinal organs may be of particular concern due to the increased potential for synergistic toxicity. More data are necessary to determine optimal dose constraints and fractionation in this setting.

The only antibody–drug conjugate with a growing number of data in combination with radiotherapy is T-DM1. The majority of existing data are from the combined use of T-DM1 with adjuvant locoregional radiotherapy of the breast or chest wall [30,31,32,33,34]. Data from the randomized controlled KATHERINE and ATEMPT trials demonstrated similar rates of radiotherapy-related toxicity when comparing trastuzumab monotherapy to T-DM1 with concurrent use during radiotherapy [31,33]. Thus, the concurrent use of T-DM1 with adjuvant radiotherapy is considered safe [35].

By contrast, the safety of T-DM1 when associated with stereotactic radiotherapy for brain metastases is debated [36]. A recent systematic review found rates of grade 2+ and grade 3+ radiation necrosis of 37% and 17% for intracranial radiotherapy with T-DM1, respectively [37]. However, this was based exclusively on retrospective case series with < 50 patients. Furthermore, there was significant heterogeneity between studies.

According to our systematic review, there is a limited number of data on the concomitant use of SG and radiotherapy. The recent publication by Lebow et al. suggests that the concurrent use of stereotactic radiotherapy and ADCs may increase the risk of radiation necrosis after ablative doses with stereotactic techniques [19]. However, the majority of patients in this analysis had received T-DM1, where an elevated risk of radiation necrosis had already been described [37]. The authors did not find a significant difference in the incidence of radiation necrosis between the different ADCs and no increased risk in patients receiving SG; however, additional data are needed due to the limited number of patients. Furthermore, the definition of concurrent use was very broad. Symptomatic radiation necrosis was more frequent in patients with re-irradiation.

Current ongoing clinical trials (SASCIA/GBG 102, ASCENT-05, ASCENT-03, ASCENT-04, ASCENT-07) on the use of SG in various settings will not provide further insight into the simultaneous use of SG and radiotherapy, as radiotherapy must be completed before enrolment in the trial.

Our retrospective study with 17 patients represents the only available multi-center evidence on the concomitant administration of SG and radiotherapy. This underlines the importance and the need for high-quality registry studies to systematically record real-world experience with new therapies. Our experience with 17 patients who received treatment with SG and radiation suggests that concomitant use is safe and was well tolerated by patients. Furthermore, the comparison between simultaneous and sequential use of SG and radiotherapy in our patient cases showed no increase in moderate-to-severe toxicity. No unexpected toxicities were observed. Grade 3 toxicity potentially attributable to radiotherapy or the combination of radiotherapy and SG occurred only in patients with prior irradiation and field overlap. The cases described here were all heavily pretreated patients with metastatic TNBC. In the practical management of SG, we have learned that it is useful to treat patients with G-CSF as primary prophylaxis to avoid severe neutropenia and associated risks.

Due to the recent approval of SG for metastatic hormone receptor-positive HER2-negative breast cancer after endocrine and at least two further systemic therapies, this has implications beyond TNBC. While toxicity is expected to be similar, response rates may differ according to tumor biology.

The main limitation of this retrospective analysis is the relatively small number of clinical cases, as well as the heterogeneity of clinical situations. While this reflects real-world practice from six German centers, it is difficult to draw meaningful conclusions due to the heterogeneity of patient characteristics and treatment. Therefore, a formal statistical analysis is not feasible. The follow-up of the reported patient cases was short, which is mostly related to the poor prognosis of patients with TNBC in advanced treatment lines. Thus, the potential risk of late toxicity arising from the combination of radiotherapy and SG cannot be assessed. There is wide heterogeneity in the definition of concomitant, simultaneous and sequential treatment with radiotherapy and systemic agents. We followed a pragmatic approach by including patients who received radiotherapy within 90 days of SG. Furthermore, we defined simultaneous treatment as SG administration during the radiotherapy course due to the limited half-life of the payload. Based on the pharmacokinetics of the antibody component, interactions between RT and SG may occur during a wider time window. Further research should address this area of uncertainty.

## 5. Conclusions

Our analysis provides the first clinical data regarding the combination of radiotherapy and SG. In the presented cases, this combination appears to be safe, and promising local response rates were observed despite extensive prior treatment. In clinical practice, an interdisciplinary discussion to assess the potential benefits and risks should be established on a case-by-case basis. Cases involving radiotherapy to areas with prior irradiation should be approached with caution. Whenever the risk of radiotherapy-related toxicity is elevated, the simultaneous use of radiotherapy and SG should be avoided, as long as this is justifiable from an oncological perspective. Furthermore, dose to normal tissues should be minimized. This analysis can only be regarded as a first step and should encourage other clinicians to analyze additional cases to provide further insight into this important clinical question.

## Figures and Tables

**Table 1:** List of databases and search terms.

Databases	Search Algorithm
Ovid MEDLINE, Journals@Ovid BIOSIS Previews and Citations Daily between 1946 and 1 February 2024 and Embase between 1974 and 1 February 2024	ALL = [trodelvy OR sacituzumab govitecan OR isactuzumab govitecan OR anti-TROP-2-SN-38 OR GS-0132 OR GS0132 OR “GS 0132” OR hRS7-CL2-SN-38 OR HRS7-CL2-SN-38 OR HRS7-SN-38 OR HRS7-SN38 OR UMMU-132 OR UMMU132 OR “UMMU 132” OR IMMU-132 OR IMMU132 OR “IMMU 132” OR RS7--SN38 OR RS&SN38] AND [radiation OR irradiated OR radiotherapy OR irradiation]

**Table 2:** Patient and treatment characteristics. SG = Sacituzumab govitecan.

	Total (N = 17)
Median age, years (range)	48 (33–73)
Median number of prior treatment lines	2.5 (1–6)
Median number SG cycles (range)	5 (2–26.5)
Median total dose, Gy (range)	36 (8–50.4)
Median dose per fraction (range)	3 (1.8–20)
Radiotherapy technique *, number of patients (%)	
Intensity-modulated radiotherapy	13 (38.2)
Electrons	8 (23.5)
Stereotactic radiotherapy	7 (20.6)
3D-conformal radiotherapy	4 (11.8)
Unknown	2 (5.9)
Radiotherapy target volume *, number of targets (%)	
Lymph node metastases	9 (26.5)
Brain metastases	8 (23.5)
Skin metastases	8 (23.5)
Bone metastases	6 (17.6)
Breast/thoracic wall	2 (5.9)
Lung metastasis	1 (2.9)
Target volumes with re-irradiation **	12
Number of patients with simultaneous SG (%)	7 (41.1)

* Data refer to individual radiotherapy courses/target volumes. ** At least partial overlap between irradiated volumes.

## Data Availability

The raw data supporting the conclusions of this article will be made available by the authors on request.

## Supplementary Materials

The following supporting information can be downloaded at: https://www.mdpi.com/article/10.3390/cancers16091649/s1, Table S1: Population, Intervention, Control, Outcome (PICO) criteria; Table S2: Preferred Reporting Items for Systematic Reviews and Meta-Analyses (PRISMA) Checklist; Table S3: Additional patient and treatment characteristics.
